# Emergency Department Boarding of Mechanically Ventilated Patients

**DOI:** 10.7759/cureus.23990

**Published:** 2022-04-09

**Authors:** Ahmed Mihdhar O Saggaf, Abdullah Mugharbel, Abdulrahman Aboalola, Albarra Mulla, Meshal Alasiri, Muhannad Alabbasi, Abdullah Bakhsh

**Affiliations:** 1 Department of Emergency Medicine, King Abdulaziz University, Jeddah, SAU; 2 Faculty of Medicine, King Abdulaziz University, Jeddah, SAU; 3 Family Medicine, King Abdulaziz University Faculty of Medicine, Jeddah, SAU

**Keywords:** boarding, critical care, saudi arabia, mechanical ventilation, emergency department boarding

## Abstract

Background and objective

The boarding of critically ill patients in the emergency department (ED) has been on the rise over the past few years. Emergency physicians now frequently encounter critically ill patients who require rapid resuscitation and stabilization and they provide extended care in the ED. This study aimed to evaluate the association between the boarding duration of mechanically ventilated patients in the ED and outcomes in such patients.

Methods

This was a retrospective study conducted during the period 2018-2019 at an academic institution; it included adult patients who were mechanically ventilated, requiring and awaiting admission to the ICU from the ED.

Results

We included a total of 388 out of 537 patients in the analysis. Patients were stratified into three groups as follows: 93 (24%) were admitted to the ICU within six hours; 126 (32.5%) were admitted to the ICU within 6-24 hours; and 169 (43.6%) were admitted to the ICU after 24 hours. Patients admitted to the ICU within six hours were significantly younger; the mean age of the patients was 55 ± 16.30 years in group 1, 61.96 ± 17.73 years in group 2, and 62.65 ± 16.62 years in group 3 (p=0.001). The ICU mortality in group 1 was lower than in other groups, and mortality increased with increasing boarding time [28 (30.1%), 51 (40.5%), 79 (46.7%), respectively, p=0.032]. Boarding time in the ED was associated with an increased risk of ICU mortality in group 3 compared with group 1 (0.1664 ± 0.063, p=0.009). The logistic regression analysis showed higher mortality rates in groups 2 [adjusted odds ratio: 3.29; 95% confidence interval (CI): 1.95-5.55, p<0.01] and 3 (adjusted odds ratio: 1.98; 95% CI: 1.17-3.35, p=0.01).

Conclusion

Based on our findings from this small-sample, single-center study, ED boarding of mechanically ventilated patients is associated with higher ICU mortality rates.

## Introduction

In healthcare settings, emergency departments (EDs) are currently providing more and more critical care procedures and management traditionally associated with ICU care [1]. The rate of patients requiring critical care in EDs increased by 80% between 2004 and 2014 [1]. Moreover, the number of patients intubated in the ED increased by 16% during this period [1]. The increasing prevalence of conditions such as chronic obstructive pulmonary disease (COPD), hypertension, diabetes, sepsis, and trauma has led to a rise in critically ill or injured patients presenting to the ED [1]. The American College of Emergency Physicians (ACEP) defines ED boarding as a situation where patients remain in the ED after they have been admitted to an admitting service at the facility but have not yet been transferred to an inpatient unit [2]. Current literature suggests that boarding is common for critically ill or injured patients in the ED [2]. Several studies have demonstrated that increasing ED boarding time is associated with worse outcomes for patients requiring ICU-level care [3]. These include increased duration of mechanical ventilation, longer ICU length of stay, higher mortality, worsening organ dysfunction, and poor neurologic recovery in patients presenting with a stroke [4,5,6]. Studies have found that critically ill patients experiencing boarding for more than six hours carry a higher risk of inpatient mortality [7,8]. In the United States, 33% of ICU admissions from the ED have an ED length of stay of more than six hours.

No widely accepted consensus exists on defining time thresholds for ED boarding [1]. This has led researchers to develop their own, varying time thresholds. Several studies have defined a time threshold a priori of greater than two, four, or six hours [4,7,9,10]. The DELAY-ED study group has identified six hours in the ED (from triage) as the definition of ED boarding. This was based on their observation that adverse outcomes among admitted critically ill patients are more frequent with an ED stay of longer than six hours [7]. Al-Qahtani et al. retrospectively reviewed critically ill patients boarding in the ED at a 900-bed academic hospital in Riyadh [11]. This study showed that increased ED boarding was associated with increased in-hospital mortality. Moreover, patient days on mechanical ventilators increased with increased ED boarding.

The purpose of our study was to investigate the association between ED boarding duration of mechanically ventilated patients and patient outcomes including hospital length of stay, ICU length of stay, and ICU mortality.

## Materials and methods

Study design

This was a retrospective cohort study of mechanically ventilated patients in the ED who were awaiting ICU admission. This study was approved by the Research and Ethics Committee at King Abdulaziz University (reference no. 700-18). We identified all patients requiring mechanical ventilation in the ED via the International Classification of Diseases, Tenth Revision (ICD-10) procedure code 0BH17EZ. Patients were included in the analysis if they met the study inclusion criteria.

Study setting

The setting was a single-center tertiary care academic ED with an accredited emergency medicine residency training program. The annual ED census at this facility is 60,000 visits. We included all patients who were intubated in the ED between 2018 and 2019. We included patients who were (1) located in the ED, (2) required admission to the ICU, and (3) mechanically ventilated. We excluded patients aged younger than 18 years, pregnant women, prisoners, and patients not requiring mechanical ventilation in the ED.

Mechanical ventilation

Mechanical ventilation is the standard care within the department of emergency medicine to initiate a volume-controlled, lung-protective ventilation strategy, which entails the following: (1) tidal volume of 6-8 mL/kg predicted body weight and (2) target plateau pressure of less than 30 cmH_2_O. Other parameters are adjusted based on the clinician's judgment. Respiratory therapists frequently monitor and adjust ventilator settings according to clinician orders. The department at this facility uses the Servo-i mechanical ventilator (Getinge AB, Göteborg, Sweden) for patients requiring mechanical ventilation. Patients in the ED undergo rapid-sequence intubation as per an established protocol.

Statistical analysis

Continuous data were expressed as means and standard deviations (SD) and compared using the students' t-test. Categorical data were expressed in percentage points and compared using the chi-square test. We used the analysis of variance (ANOVA) test to compare the mean values of the three groups (less than six hours, 6-24 hours, and more than 24 hours). We employed binary logistic regression analysis to examine the independent association of boarding time (less than six hours, 6-24 hours, and more than 24 hours) with ICU mortality rates while adjusting for confounding variables such as age, gender, admitting diagnosis, hemodynamic parameters, hospital length of stay, laboratory values, and the Acute Physiology and Chronic Health Evaluation (APACHE II) scores. All analyses were performed using SPSS Statistics version 21 (IBM, Armonk, NY). Statistical significance was set at an alpha of less than 0.05.

## Results

Baseline characteristics

During the study period, we screened a total of 537 patients admitted to the ICU from the ED. Of those, only 388 (72.2%) patients were mechanically ventilated and required admission to the ICU. We stratified patients into three groups based on boarding time in the ED: group 1 had a boarding time of less than six hours (24%), group 2 had a boarding time of 6-24 hours (32.5%), and group 3 were boarding for more than 24 hours (43.6%). Baseline characteristics of the patients are shown in Table 1. Patients in group 1 were significantly younger (55 ± 16.30 years in group 1, 61.96 ± 17.73 years in group 2, and 62.65 ± 16.62 years in group 3; p=0.001). The admitting diagnosis mainly included cardiovascular conditions, followed by respiratory, gastrointestinal, sepsis or septic shock, and other medical conditions. Cardiovascular reasons for admission were significantly higher in group 1 than the other groups [41 (44.1%) in group 1, 26 (20.6%) in group 2, 51 (30.2%) in group 3; p=0.001]. Also, sepsis and septic shock were significantly more in group 2 than in the other groups [seven (7.5%), 26 (20.6%), 29 (17.2%), respectively; p=0.028].

With regard to laboratory findings, patients in group 1 had a statistically higher hematocrit level than groups 2 and 3 (34.41 ± 10.36% in group 1, 31.17 ± 9.20% in group 2, and 31.49 ± 8.04% in group 3; p=0.02). In terms of hemodynamic parameters, both mean arterial pressure (MAP) and systolic blood pressure (SBP) were statistically lower in group 1 than in groups 2 and 3, as shown in Table 1.

**Table 1:** Baseline characteristics according to ED boarding time ED: emergency department; MAP: mean arterial pressure; SD: standard deviation

Variable	Group 1 (<6 hours), n=93	Group 2 (6–24 hours), n=126	Group 3 (>24 hours), n=169	P-value
Age, years, mean ± SD	55 ± 16.30	61.96 ± 17.73	62.65 ± 16.62	0.001
Gender, n (%)				0.112
Female	30 (32.3)	52 (41.3)	77 (45.6)	
Male	63 (67.7)	74 (58.7)	92 (54.4)	
Admission category, n (%)				
Cardiovascular	45 (48.4)	37 (29.4)	67 (39.6)	0.01
Respiratory	12 (12.9)	26 (20.6)	34 (20.1)	0.274
Liver	1 (1.1)	3 (2.4)	7 (4.1)	0.337
Gastrointestinal	8 (8.6)	9 (7.1)	7 (4.1)	0.310
Intracerebral hemorrhage	10 (10.8)	6 (4.8)	13 (7.7)	0.248
Metabolic	7 (7.5)	3 (2.4)	21 (12.4)	0.466
Sepsis	7 (7.5)	26 (20.6)	29 (17.2)	0.02
Laboratory values, mean ± SD				
Sodium, mEq/L	138.14 ± 15.94	137.48 ± 14.51	139.55 ± 9.30	0.394
Potassium, mmol/L	4.27 ± 1.84	4.84 ± 8.90	4.75 ± 10.74	0.889
Creatinine, μmol/L	181.87 ± 229.92	237.06 ± 275.71	238.64 ± 229.78	0.182
Hematocrit, %	34.41 ± 10.36	31.17 ± 9.20	31.49 ± 8.04	0.024
White blood cell count, x 10^9^/L	13.88 ± 6.74	14.22 ± 12.05	12.42 ± 7.09	0.210
Hemodynamic parameters, mean ± SD				
Diastolic blood pressure, mmHg	69.85 ± 20.68	66.75 ± 16.90	70.31 ± 16.57	0.361
Systolic blood pressure, mmHg	115.43 ± 23.82	121.49 ± 23.01	126.29 ± 22.52	0.019
MAP, mmHg	44.31 ± 44.42	51.94 ± 43.71	63.70 ± 42.71	0.002
Canadian Triage and Acuity Scale, n (%)				0.295
CTAS 1	16 (17.2)	12 (9.5)	10 (5.9)	
CTAS 2	61 (65.6)	89 (70.6)	128 (75.7)	
CTAS 3	13 (14.0)	22 (17.5)	30 (17.8)	

Outcomes

Hospital length of stay (in hours) was statistically longer in group 3 than in groups 1 and 2 (360.89 ± 558.36 hours in group 1, 385.95 ± 404.74 hours in group 2, and 549.46 ± 626.72 hours in group 3; p<0.01). Moreover, ICU mortality was statistically higher in group 3 than in the other groups (30.1% in group 1, 40.5% in group 2, and 46.7% in group 3; p=0.03). However, ICU length of stay was not statistically different between the groups. Table 2 displays the results of outcomes associated with ED boarding.

**Table 2:** Outcomes according to ED boarding time ED: emergency department; ICU: intensive care unit; LOS: length of stay; SD: standard deviation

Outcome	Group 1 (<6 hours), n=93	Group 2 (6–24 hours), n=126	Group 3 (>24 hours), n=169	P-value
ICU mortality, n (%)	28 (30.1%)	51 (40.5%)	79 (46.7%)	0.032
ICU LOS, hours, mean ± SD	346 ± 559	342 ± 391	420 ± 574	0.349
Hospital LOS, hours, mean ± SD	360 ± 558	385 ± 404	549 ± 626	0.008

Logistic regression analysis demonstrated that ICU mortality was higher with increased ED boarding time of more than 24 hours (group 3) (adjusted odds ratio: 1.98; 95% confidence interval (CI): 1.17-3.35, p=0.01). Furthermore, ICU mortality was higher with increased ED boarding time of more than six hours but less than 24 hours (group 2) (adjusted odds ratio: 3.29; 95% CI: 1.95-5.55, p<0.01), as shown in Table 3. The remaining outcomes did not show statistical significance after adjusting for confounding factors. Table 3 shows the logistic regression analysis results (Nagelkerke value: 0.023).

**Table 3:** Logistic regression analysis examining the association between ED boarding time and ICU mortality aOR: adjusted odds ratio; CI: confidence interval; ED: emergency department; ICU: intensive care unit

ED boarding time	ICU mortality (aOR; 95% CI)	P-value
ED boarding of more than 6 hours but less than 24 hours	3.29; 1.95-5.55	<0.01
ED boarding of more than 24 hours	1.98; 1.17-3.35	0.01

## Discussion

Our findings shed more light on the practice of boarding critically ill patients in the ED. The current study confirms that ED boarding is detrimental to patient care. Previous studies have shown that critically ill patients have better results when managed in ICUs [12,13,14]. Critically ill patients show improved outcomes when resuscitation starts quickly [15]. Although emergency physicians are trained to provide optimal management during the resuscitation of critically ill patients, the ED is neither designed nor adequately staffed to provide extended care to critically ill patients [11].

A large, United States-based study examined approximately 50,000 patients from 120 ICUs and found that delayed admission was associated with increased hospital length of stay and higher ICU mortality rates [7,11]. An Australian study revealed examined outcomes of critically ill patients admitted directly to an ICU (direct group) versus patients admitted to the ICU within 24 hours of ward admission from the ED (delayed group). They concluded that patients in the delayed group had a significantly higher mortality rate compared to the direct group [16]. These studies are in concordance with the findings seen in our study. Many factors could be associated with increased mortality in ED-boarding patients. One possible explanation is that the busy, fast-paced nature of the ED may not allow physicians and nurses to provide the focused one-on-one care that a critically ill patient requires in terms of quality medical care [12]. Furthermore, critically ill patients need continuous and close involvement by dedicated physicians and nurses. Unfortunately, most EDs are not designed for providing such care. To overcome this challenge, Gunnerson et al. created an ED-based ICU care delivery model that provides dedicated space and resources combined with nurse and physician staffing based on traditional inpatient ICU care [3]. This was designed to optimize time-sensitive diagnosis and treatment for patients who are critically ill or injured [3]. This strategy was associated with significant reductions in mortality and inpatient ICU admissions [3].

On the other hand, three studies investigating ED patients admitted to ICUs found no association between ED boarding and in-hospital mortality [17,18,19]. There are various possible explanations for these negative results. The early treatment provided by the ED staff to critical patients may have attenuated the impact of ED boarding, suggesting that the timely treatment could be the factor that defined mortality as compared to the boarding time [17]. The standardization of care for ED patients with sepsis, thanks to the widespread use of sepsis protocols, may have contributed to reducing deaths among septic ED patients [18]. The implementation of countermeasures, such as prioritizing the perceived sickest ICU patients for available beds, may have mitigated the potential mortality effects of boarding for ED patients who were admitted to ICUs [19]. These results confirmed previous studies showing that the ICU admission delay had no effect on survival in critically ill patients [20].

To improve care and reduce mortality among critically ill patients boarding in the ED, administrators should explore all options to alleviate the drawbacks of ED boarding, either by emphasizing the importance of making ICU beds available for ED-boarding patients or endorsing the design of an ED-based ICU with careful collaboration between departments of critical care medicine, emergency medicine, and other key stakeholders.

Limitations

Our study has several limitations. Firstly, this was a single-center study with small sample size, and hence our findings may not be generalizable to the broader population. Second, since the study was observational in nature, it may have failed to capture all possible confounding factors such as mechanical ventilator settings and blood gas analyses. Third, there was a statistically significant difference between the three groups in terms of age, and it has had an effect on our results. Finally, we included broad diagnostic categories rather than specific conditions. We did, however, attempt to reduce the effect of confounding by introducing a logistic regression analysis.

## Conclusions

Our findings demonstrate that longer ED boarding of mechanically ventilated patients (more than six hours) is associated with longer hospital length of stay and higher ICU mortality rates. Moreover, ED boarding for more than six hours predicted higher ICU mortality rates, after adjusting for confounding factors. Institutions should focus on either rapidly admitting critically ill patients to the ICU, within six hours, or designing an ED-based ICU for providing more optimal and extended care to critically ill patients.
